# Femtosecond Thermal and Nonthermal Hot Electron Tunneling Inside a Photoexcited Tunnel Junction

**DOI:** 10.1021/acsnano.2c04846

**Published:** 2022-08-26

**Authors:** Natalia Martín Sabanés, Faruk Krecinic, Takashi Kumagai, Fabian Schulz, Martin Wolf, Melanie Müller

**Affiliations:** † Department of Physical Chemistry, Fritz Haber Institute of the Max Planck Society, Faradayweg 4-6, 14195Berlin, Germany; ‡ IMDEA Nanoscience, Faraday 9, 28049Madrid, Spain; § Center for Mesoscopic Sciences, Institute for Molecular Science, 444-8585Okazaki, Japan

**Keywords:** hot electron tunneling, ultrafast scanning tunneling microscopy, femtosecond electron dynamics, THz near-field sampling, lightwave-induced tunneling, two-temperature model

## Abstract

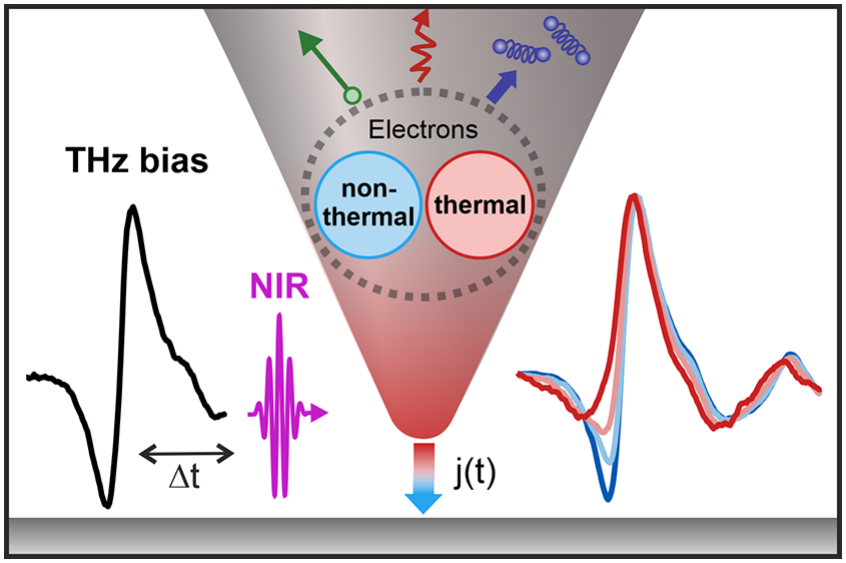

Efficient operation of electronic nanodevices at ultrafast speeds requires understanding and control of the currents generated by femtosecond bursts of light. Ultrafast laser-induced currents in metallic nanojunctions can originate from photoassisted hot electron tunneling or lightwave-induced tunneling. Both processes can drive localized photocurrents inside a scanning tunneling microscope (STM) on femto- to attosecond time scales, enabling ultrafast STM with atomic spatial resolution. Femtosecond laser excitation of a metallic nanojunction, however, also leads to the formation of a transient thermalized electron distribution, but the tunneling of thermalized hot electrons on time scales faster than electron–lattice equilibration is not well understood. Here, we investigate ultrafast electronic heating and transient thermionic tunneling inside a metallic photoexcited tunnel junction and its role in the generation of ultrafast photocurrents in STM. Phase-resolved sampling of broadband terahertz (THz) pulses via the THz-field-induced modulation of ultrafast photocurrents allows us to probe the electronic temperature evolution inside the STM tip and to observe the competition between instantaneous and delayed tunneling due to nonthermal and thermal hot electron distributions in real time. Our results reveal the pronounced nonthermal character of photoinduced hot electron tunneling and provide a detailed microscopic understanding of hot electron dynamics inside a laser-excited tunnel junction.

Hot carriers harvested in solids through optical excitation can drive a wide range of physical and chemical processes, the understanding of which lies at the heart of hot carrier science and technology.^1^ The ultrafast dynamics of photoexcited hot carriers are intimately related to optoelectronic nanodevice operation. A particular example is laser-excited ultrafast scanning tunneling microscopy (STM), which pursues the ultimate goal to image the ultrafast dynamics of individual atoms and molecules on surfaces with simultaneous Angstrom spatial and femtosecond temporal resolution. Recent advances in combining ultrashort optical or terahertz (THz) pulses with low-temperature STM demonstrated the potential of light-driven STM to reach that goal.^2−6^ Attaining ultrahigh spatiotemporal resolution in STM requires the generation of ultrafast, atomically localized tunneling currents. In the weak excitation regime, photoassisted tunneling of hot electrons below the vacuum barrier gives rise to localized ultrafast currents that can be used for imaging in photon-driven STM.^6−8^ In contrast, lightwave-driven STM operates in the strong-field regime, where an intense localized light field of several V/nm modulates the junction barrier and, hence, the tunneling current on subcycle time scales.^2,4,5,9−13^ The two regimes are commonly distinguished by the Keldysh parameter, , relating the work function of the material, , to the photon energy, , and the light field, .^14^

Both processes are distinctly different in terms of the involved electronic excitation: Whereas photoassisted tunneling employs the tunneling of “hot” but nonthermal electrons, lightwave-driven STM is mediated by adiabatic tunneling of thermal but “cold” electrons directly from the Fermi level. Herein, the terms “hot” and “cold” describe whether there is excess energy (e.g., via absorption) stored in the electronic system, whereas the terms “thermal” and “nonthermal” state whether the electronic distribution follows Fermi–Dirac statistics. Focusing ultrashort laser pulses on a metal gives rise to light absorption and the generation of energetic, nonthermal electrons, which rapidly redistribute their energy via electron–electron and electron–phonon scattering, leading to the formation of a thermalized carrier distribution on the 10–100 fs time scale. Hot electrons from both thermal and nonthermal distributions can tunnel through the potential barrier in STM, though with different time scales and energy distributions of the respective tunneling currents. Although the ultrafast dynamics and thermalization of hot electrons in metals has been intensively investigated,^15,16^ the transient tunneling of thermalized hot electrons is rarely considered as source for ultrafast photocurrents in STM. However, ultrafast thermionic tunneling should coexist with both photon-driven and lightwave-driven tunneling currents and thus accompanies any pulsed laser-driven STM. Moreover, the dynamics of hot electrons determine the initial step of laser heating of STM tips illuminated by ultrashort laser pulses.^17,18^ It is thus of general interest to understand the role of hot electron dynamics for femtosecond heating and tunneling in photoexcited STM.

Transient thermionic emission has been discussed in the context of ultrafast electron guns using laser-excited nanotips.^19−22^ It describes the emission of electrons above the vacuum barrier originating from the high-energy tail of a time-dependent Fermi–Dirac carrier distribution at elevated temperatures^23,24^ and can be described by the Richardson–Dushmann equation.^25^ In contrast to freestanding tips, the nanometer-sized tip–sample gap in STM allows electrons to tunnel through the barrier at all energies down to the Fermi level; therefore, ultrafast thermionic currents can flow at much lower electron temperatures. The situation is similar to thermally enhanced field emission from a high voltage (kV) biased nanotip emitter^19,26,27^ operating at fields of >10^8^ V/m, which can be described by Murphy’s and Good’s equation for thermo-field emission.^28^ To understand thermionic tunneling on femtosecond time scales, the ultrafast evolution of the electronic temperature after laser excitation has to be known. Before electron–phonon equilibration has been achieved (>1 ps), different temperatures are assigned to the electronic and phononic subsystems. Their respective time evolutions can be described by the two-temperature model (TTM)^15,19^ or by solving Boltzmann transport equations^16,20^ for the coupled electron–phonon system. Whereas photoassisted tunneling and optical-field-driven tunneling are temporally confined to the presence of the laser pulse, thermionic tunneling can occur on time scales longer than the laser pulse duration and might lead to significantly delayed photocurrents in STM due to electron dynamics inside the tip.

Here we study the interplay between ultrafast thermal and nonthermal tunneling from a femtosecond laser-excited STM tip. By tracking the temporally delayed response of thermionic currents in real-time we can discriminate between noninstantaneous thermal and instantaneous nonthermal currents, without the need to distinguish their energy distributions as for example in photoemission experiments.^20,21,29^ Energy information could, in principle, be obtained in STM by varying the bias voltage, but the bidirectional tunneling of photocarriers between tip and sample complicates interpretation of the respective hot carrier distributions. Instead, we measure the temporal response of ultrafast thermionic currents via phase-resolved sampling of single-cycle ultrabroadband THz pulses inside the tip–sample junction.^30^ Specifically, we measure the time-dependent THz-induced change of photocurrents from the laser-excited STM tip,^30−32^ which is determined by the instantaneous THz bias  and the time evolution of the photocurrent . In the case of prompt currents, the THz-field-induced photocurrent modulation yields the tip-enhanced THz near-field waveform. In contrast, delayed photocurrents will suppress higher THz frequencies and modify the measured waveform similar to a low-pass filter that limits sampling bandwidth. Knowledge of the original instantaneous THz waveform combined with numerical simulation of the time-dependent tunneling process allows us to extract the thermionic photocurrent dynamics and relate it to the electronic temperature evolution inside the STM tip and to reveal the competition between thermal and nonthermal photocurrent contributions.

## Results and Discussion

### Phase-Resolved THz Sampling of Ultrafast Photocurrents in STM

**Figure 1:**
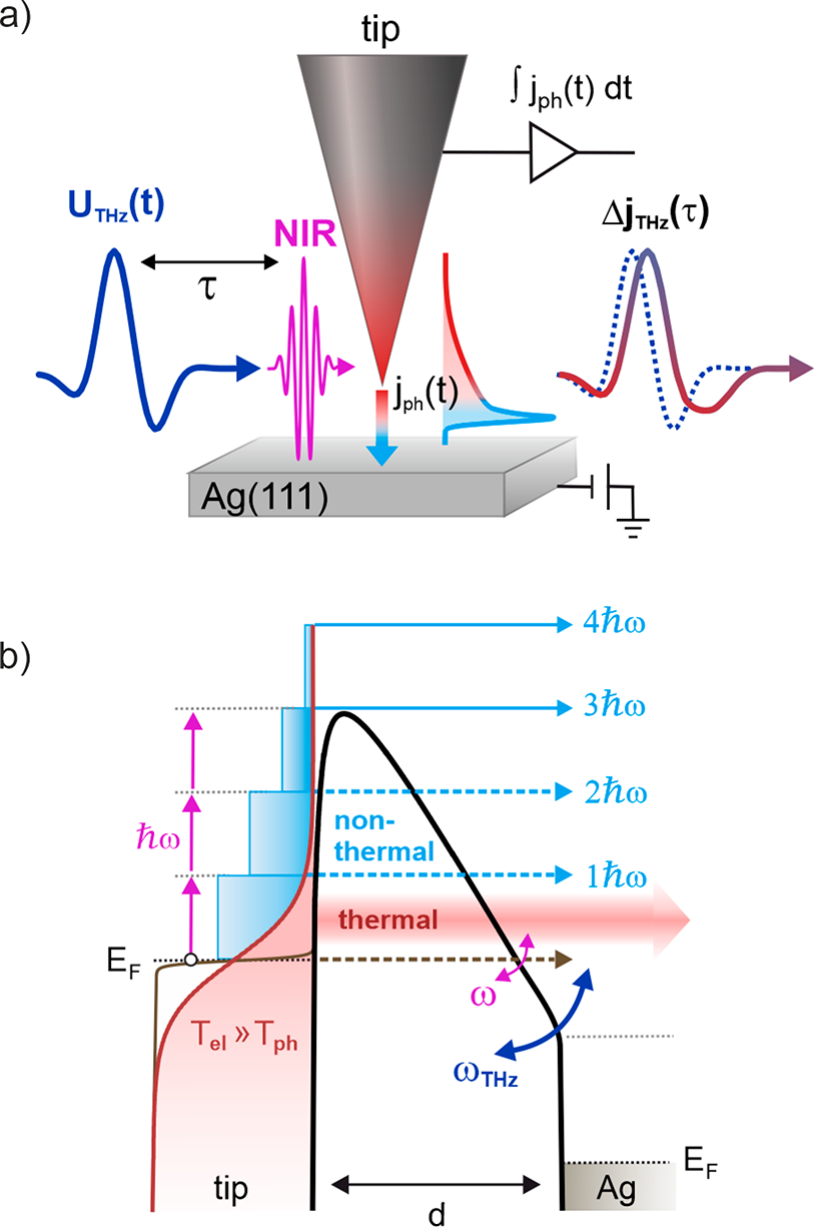
Real-time sampling of ultrafast thermionic currents in a photoexcited THz-gated STM junction. (a) A time-dependent photocurrent , which can contain fast (light blue) and slow (red) components, is excited from the STM tip by 10 fs NIR laser pulses. The time evolution of the photocurrent is probed by phase-stable single-cycle THz pulses acting as a quasi-static bias that modulates the potential barrier. A delayed photocurrent response is encoded in the measured THz waveform obtained from the THz-induced change of the photocurrent, . (b) Photocurrent channels from the laser-excited STM tip. On the time scale of the NIR pulse duration, above-barrier photoemission and photoassisted tunneling at energies  (light blue arrows) lead to nonthermal currents. At longer times >10 fs, the tunneling of thermalized hot electrons (red arrow) can lead to delayed photocurrents depending on the time evolution of the electron temperature . Strong NIR laser fields can induce suboptical-cycle tunneling from a “cold” electron distribution at the Fermi level (gray dashed arrow). The THz field modulates the potential barrier and the photocurrent yield of all channels according to their respective bias dependence and emission times.

Figure 1a shows the experimental scheme of our measurement. We excite a grounded tungsten STM tip with 800 nm near-infrared (NIR) laser pulses of 10 fs duration with peak intensities ranging from  to  W/cm^2^, which corresponds to laser powers between 0.11 and 0.36 mW under our conditions. The tip is placed above the Ag(111) sample at a distance *d*. A positive bias of  is applied to the sample to suppress photocurrents from the sample with opposite sign and to ensure unidirectional flow of photocurrents originating from the tip. Phase-stable single-cycle broadband THz pulses are focused into the STM junction at variable delay τ with respect to the NIR pulses. The quasi-instantaneous field of the tip-enhanced THz field acts as a transient bias modulating the potential barrier and hence the current on femtosecond time scales. Provided that the THz-induced change of the photocurrent, , is dominated by a process that is instantaneous on the time scale of the THz field, the THz bias can be sampled with a time resolution as determined by the NIR pulse duration. The delay-dependent  reveals the transient THz bias, and the local slope of the photocurrent–voltage curve allows us to calibrate the THz bias amplitude .^30,32^ Precise knowledge of the waveform and amplitude of the THz bias will be essential to extract the time evolution of ultrafast noninstantaneous currents photoexcited inside the metallic junction, which will lead to temporal distortions of the measured THz waveform as illustrated in Figure 1a. We point out the difference from “state-selective” THz-gated tunneling,^2^ where the resonant THz-induced tunneling into individual molecular states during the peak of a THz half-cycle yields a time resolution faster than the THz half-cycle. Due to the threshold character of the resonant tunneling process, this regime does not require detailed knowledge of the THz waveform to access subcycle dynamics. In contrast, accessing the dynamics of “nonresonant” systems (i.e., systems with a continuous variation of electronic states such as metals or semimetals) on THz-subcycle time scales demands phase-resolved sampling of the THz waveform and knowledge of the THz bias transient.

The different photocurrent mechanisms that can be operative for a photoexcited STM tip are displayed in Figure 1b. At nanometer gap sizes, photoassisted tunneling^6,7,33^ through the barrier from a nonthermal steplike electron distribution can occur at energies that are multiples of the NIR photon energy . In comparison, the tunneling of thermalized hot electrons will be energetically distributed according to the Fermi–Dirac distribution at a given electron temperature . At large gap size, electron emission above the barrier from either nonthermal multiphoton photoemission^17,22,29^ or transient thermionic emission^19^ will dominate the photocurrent. At high laser intensities, photocurrents might be generated in the strong-field regime, for which above-threshold photoemission^34−36^ and optical-field-induced tunneling^4,37−40^ have to be considered as well. The time-dependent THz field acts an additional quasi-static bias that modulates the NIR-induced photocurrent channels according to their respective dependence on the potential barrier between tip and sample. The THz bias thus selectively probes those photocurrent channels that are most sensitive to the potential barrier.

### Laser-Induced Ultrafast Electron Heating of the STM Tip

**Figure 2:**
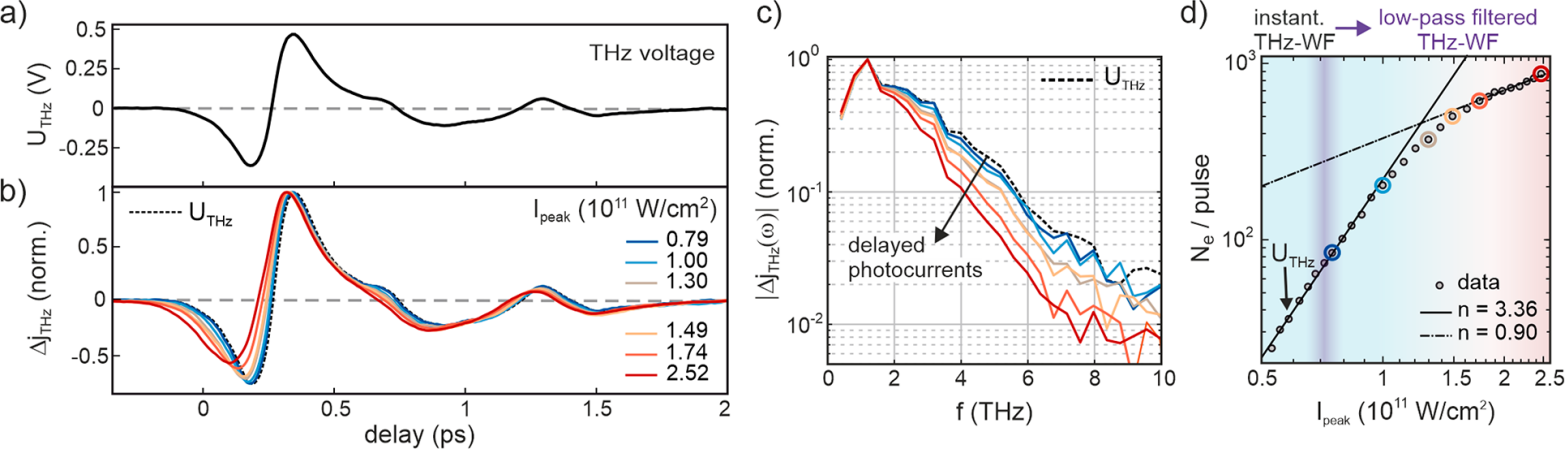
Phase-resolved THz sampling of ultrafast noninstantaneous photocurrents from the STM tip at large gap distances. (a) THz bias measured at weak optical excitation ( W/cm^2^), where quasi-instantaneous multiphoton photoemission above the barrier dominates the photocurrent. (b) THz waveforms measured at increasing NIR laser intensity, exhibiting a power-dependent distortion that resembles the characteristic of a low-pass filter, as also evident from the Fourier spectra of the waveforms plotted in (c). (d) The time-averaged photocurrent exhibits a transition from the multiphoton regime (power exponent  to the strong-field regime (. Low-pass filtered THz waveforms due to delayed photocurrents are observed in both regimes. ( μm, = 8 V).

First, we want to understand ultrafast heating for a free-standing tip. Figure 2a shows the THz bias applied to the junction of a tungsten tip and Ag(111) surface, measured for weak optical excitation far from the tunneling regime at 1 μm tip–sample distance, where the photocurrent is dominated by above-barrier multiphoton photoemission. The peak THz voltage of ∼0.4 V is small compared to the direct current (dc) bias of 8 V to ensure that the THz field only slightly perturbs the potential barrier and to prevent quasi-static THz-induced tunneling. The photocurrent–voltage curve used for calibration of the THz bias is shown in Figure S.1. Increasing the laser intensity leads to a broadening and continuous shift of the measured THz waveform , as shown in Figure 2b, where the effect is most pronounced in the first half THz cycle. The observed waveform changes are very similar to those expected from a low-pass filter, evident also from the Fourier spectra shown in Figure 2c. Figure 2d shows the laser intensity dependence of the time-averaged photocurrent measured with no THz bias applied, where the vertical purple-shaded area indicates the threshold intensity above which waveform deformations are observed for this particular tip. At low intensity, the photocurrent follows a nonlinear power scaling with effective nonlinearity , indicating that the photocurrent predominantly originates from multiphoton photoemission.^29^ At laser intensities  W/cm^2^, a significantly reduced nonlinearity of  is observed, indicating transition to the strong-field regime.^37,41,42^ The intensity at which this transition occurs is independent of the number of electrons per pulse (see Figure S.2), implying that space charge effects are not responsible for the decreasing slope. Similar data from another tip are shown in Figure S.3, where pronounced waveform changes are observed purely in the multiphoton photoemission regime. The observed waveform changes thus emerge in both the weak- and strong-field regimes of photoemission. In both regimes, the photoemission process is prompt, and the emitted current will be temporally confined to the laser pulse width. Hence, photoemission cannot explain the observed low-pass filter behavior, which requires a “slow” photocurrent contribution (i.e., a delayed carrier response). We conclude that an additional delayed photocurrent component, which coexists with instantaneous weak- and strong-field photocurrents, is responsible for the observed waveform changes. As will be discussed below, the observed waveform changes can be assigned to delayed ultrafast thermionic currents from the STM tip, whose electronic subsystem is heated on the femtosecond time scale.

### Theoretical Model

**Figure 3:**
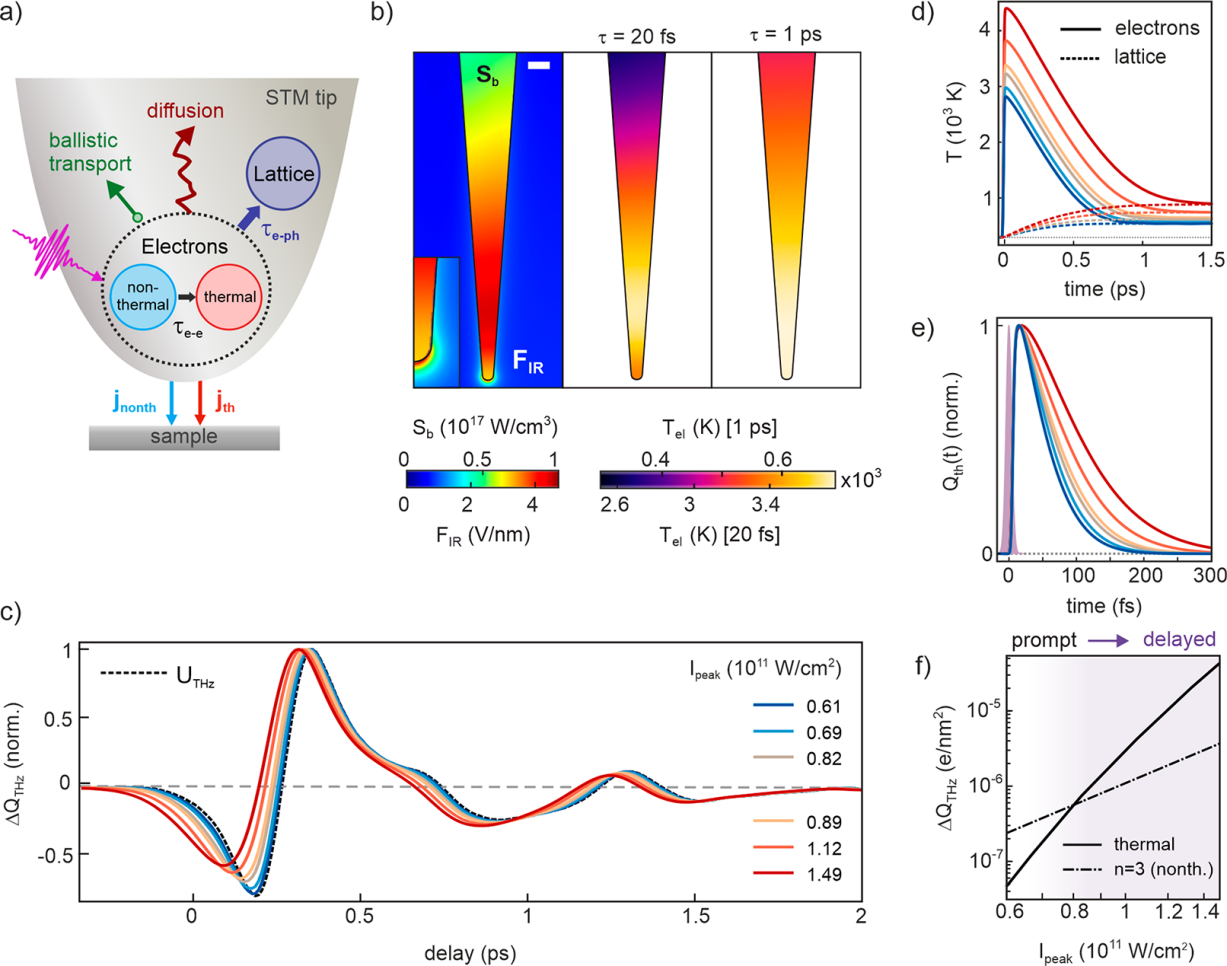
Simulation of ultrafast thermionic currents and THz waveforms from a photoexcited STM tip. (a) Microscopic picture of electron dynamics inside the tip. (b) Left panel: cycle-averaged NIR peak electric field  (outside tip) and absorbed power density  (inside tip) after ballistic distribution of the absorbed energy. Middle and right panel: Electronic temperature distribution inside the tip after fs and  ps. Scale bar is 20 nm. (c) Simulated THz waveforms retrieved from superposition of instantaneous nonthermal photocurrents and thermionic currents. The legend shows the respective incident peak intensities used in the model. (d) Time evolution of electron (solid) and lattice (dashed) temperatures and (e) the resulting thermionic current at the tip apex for the different laser intensities. The shaded area in (e) shows the intensity envelope of the NIR pulse. (f) Calculated power scaling of the THz-induced thermionic charge density  compared to that of the nonthermal charge density  through channel  at the peak of the THz pulse. ( μm,  V/nm at = 8 V,  V/nm,  nm, , ). The free parameters , , and  are adjusted to best fit the experimental THz waveforms in Figure 2b.

To understand the observed waveform changes we calculate the photocurrent from the tip and its modulation by the THz field. The microscopic picture of our model and the electron dynamics inside the metal tip are depicted in Figure 3a. The electronic system is composed of two subsystems: (i) nonthermal electrons that follow a step-like distribution and (ii) thermal electrons that follow a Fermi–Dirac distribution, as illustrated by the blue and red distributions in Figure 1b, respectively. Photoexcitation by an ultrashort laser pulse initially promotes electronic single-particle excitations, leading to a nonthermal electron distribution that thermalizes via electron–electron scattering with a rate , which takes place on time scales typically of a few  fs in transition metals.^15,16,43^ On longer time scales of  ps, electron–phonon scattering leads to energy transfer from the electronic to the phononic subsystem with a rate .^15,16^ Due to the different time scales of  and , it is reasonable to assume that electron–phonon coupling only leads to cooling of the thermal electron distribution, but it does not remove energy from the nonthermal part of the electronic system. In addition, ballistic transport and diffusion of hot electrons into the bulk can influence the electron distributions at the tip surface.^15,44^ Both nonthermal and thermal electron distributions can give rise to a photocurrent from the tip, which we write as the sum
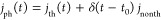
1of an ultrafast time-dependent thermionic current  and an instantaneous nonthermal current , where  is a delta function located at the laser pulse center . The separation in eq 1 and the assumption of an instantaneous nonthermal current is reasonable here considering the limited time resolution of 10s of fs determined by the finite THz bandwidth, which is insufficient to resolve the electron thermalization process via electron–electron scattering.

The ultrafast thermionic current through the THz-modulated potential barrier is given by the total charge  emitted per unit area per pulse due to a thermalized hot electron distribution,^28,45^
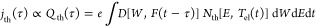
2where  is the delay between THz and NIR pulse, is the combined dc and THz electric field, and  is the time-dependent transmission probability, where  is the kinetic energy of the electron in the positive *z*-direction.  is the time-dependent thermal electron occupation at energy  and temperature  which follows the Fermi–Dirac distribution
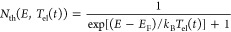
3

The THz-modulated transmission probability  is calculated by solving the one-dimensional Schrödinger equation
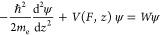
4for the time-dependent quasi-static potential
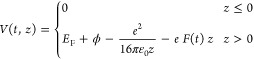
5where , , and  are the reduced Planck constant, vacuum permittivity, and electron mass, respectively,  is the Fermi level,  is the work function of the tip, and the third term in eq 5 describes the effect of image charges. Due to the high positive sample bias we can neglect current contributions originating from the sample in the present calculations. The time evolution of the electronic temperature  is obtained by solving the two-temperature model (TTM) in three dimensions inside the tip including ballistic and diffusive electron transport using COMSOL Multiphysics (details are described in section 4 of the Supporting Information). The TTM source term  for electronic heating is obtained from the spatially inhomogeneous absorbed power density, convoluted with the mean free path of electrons in tungsten^46^ to account for the initial fast redistribution of energy due to ballistic transport. The left panel in Figure 3b shows the spatial profile of  inside the tip after ballistic energy redistribution, which on the nanometric scale of the tip occurs instantaneously on the time scale of the THz field. For a given tip–sample geometry, we obtain the ultrafast electronic temperature evolution, the locally enhanced optical field as well as the dc field along the surface of the tip using COMSOL simulations. The middle and right panel in Figure 3b show the electronic temperature distribution inside the tip at  fs and  ps, respectively.  initially resembles the profile of  but becomes blurred on longer time scales due to diffusive hot carrier transport. Finally, the experimental calibration of the THz bias allows us to fix the ratio of dc and THz field. As we know the waveform of the THz bias, we can thus calculate the THz-induced change of the ultrafast thermionic current by calculating the emitted charge  in the presence of the instantaneous THz field using eq 2.

The quasi-instantaneous photocurrent due to nonthermal electrons can be written as a superposition of currents through each multiphoton channel *n*
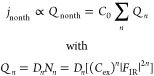
6where *Q_n_* is the charge emitted per unit area per pulse through channel *n*,  is the tip-enhanced laser field,  is a constant scaling factor, and  and  are the electron density and transmission probability at the surface at energy  with , respectively. The excitation constant  is a free parameter whose dependence on *n* accounts for the decreasing probability for excitation into higher multiphoton channels. However, the relative contribution from higher channels increases with increasing laser intensity as expected due to . Equation 6 thus follows the intensity scaling and *n*-dependent excitation probability expected for multiphoton absorption in the perturbative regime.^47^ The THz-induced change of the nonthermal charge density, , is calculated from eq 6 taking into account the time dependence of the transmission probability  due to the instantaneous THz field at the tip surface. Finally, the simulated waveforms are obtained from the sum . The free parameters of our model are the incident laser intensity, the constants  and , and the tip–sample geometry including the gap distance .

At high laser fields approaching  ∼ 1, above-threshold photoemission becomes significant. Yet, as those channels are relatively insensitive to the potential barrier and hence the THz bias, we neglect them here and restrict our calculations to channels below or close to the top of the barrier ( in our case). At even higher laser fields, entering the optical tunneling regime at , the laser field is significantly stronger than the applied THz and dc fields. Hence, optical tunneling should be almost unaffected by the weak THz fields applied here; therefore, the THz-induced change of optical-field-driven tunneling currents is expected to be very small. We simulated NIR-lightwave-tunneling from the THz-biased tip by adding the NIR laser field to the total field  in eq 5. The results confirmed that optical tunneling contributes insignificantly to the THz-induced change of the photocurrent due to its low bias sensitivity in the parameter range used here. We can thus neglect strong-field effects and optical tunneling in our waveform simulations. We further note that the measured photocurrent is NIR-cycle-averaged and integrated over energy. In contrast to energy-resolved measurements, which record the final kinetic energy of the photoelectrons after propagating through the oscillating THz field,^31^ our measurement is not sensitive to electron propagation but only to the instantaneous THz field at the tip surface. Since , we can also neglect THz streaking of photoelectrons back into the tip.

### Simulation of Ultrafast Thermionic Currents and THz Waveforms from a Photoexcited STM Tip

Figure 3c shows calculated THz waveforms obtained at the apex of a tip with work function  eV, tip radius  nm, and shaft opening angle  (a SEM image of the STM tip used in this work is shown in Figure S.5). The simulations with incident laser intensities close to those estimated from our experimental conditions reproduce very well the measured waveform broadening and temporal shift. The exact laser intensities required to observe electronic heating in the simulated waveforms vary with tip size and shape, which is consistent with the experimental variation of the laser power we require to observe waveform deformations for different tip conditions. The corresponding time dependence of the electron temperature and thermionic current are shown in Figure 3d,e, respectively. Electronic temperatures of several 1000 K are reached inside the tip on ultrafast time scales due to the small heat capacity of electrons in metals.^48^ It is important to note that thermionic currents alone do not sufficiently describe the waveforms, especially at low laser intensities at which  does not yield the original THz waveform (pure “thermionic waveforms” are shown in Figure S.4.2). To reproduce the laser power dependence of the waveforms in Figure 2b precisely, the combination of thermal and nonthermal currents is required. Whereas nonthermal currents, at far distances predominantly emitted through , dominate and reproduce the original THz waveform at low laser intensities, delayed thermionic currents are responsible for the waveform distortions at higher laser intensities. Figure 3f shows the calculated power scaling of the THz-induced change of the thermionic charge density  compared to the power scaling of the nonthermal charge density  through channel . The slope of  is much steeper than that of  and continuously decreases at higher laser intensities, as opposed to the constant slope  of the nonthermal photocurrent. Whereas the scaling of  is determined solely by the excitation into states at , which increases as , the power scaling of  is determined by the increasing electron temperature and its decreasing sensitivity to the barrier, and hence THz field, at high . Overall, the relative contribution of  is insignificant at low intensities, but it increases rapidly with increasing laser intensity. It has been discussed previously that noninteger nonlinearities, which are frequently measured for photoemission from nanotips, can be caused by long-lived hot electron distributions inside the tip,^49,50^ which is supported by our results. We note that we can exclude that the delayed currents originate from laser-driven rescattering^51^ and delayed re-emission of electrons from the tip,^21^ as the waveform distortions are observed also (and for some tips solely) in the weak-field regime, in which laser-driven electron scattering is negligible (section S3 of the Supporting Information). The results in Figures 2 and 3 thus confirm that femtosecond laser heating of the STM tip can lead to ultrafast thermal currents persisting on time scales of several 100 fs (i.e., much longer than the exciting laser pulse duration).

### Gap Size Dependence

**Figure 4:**
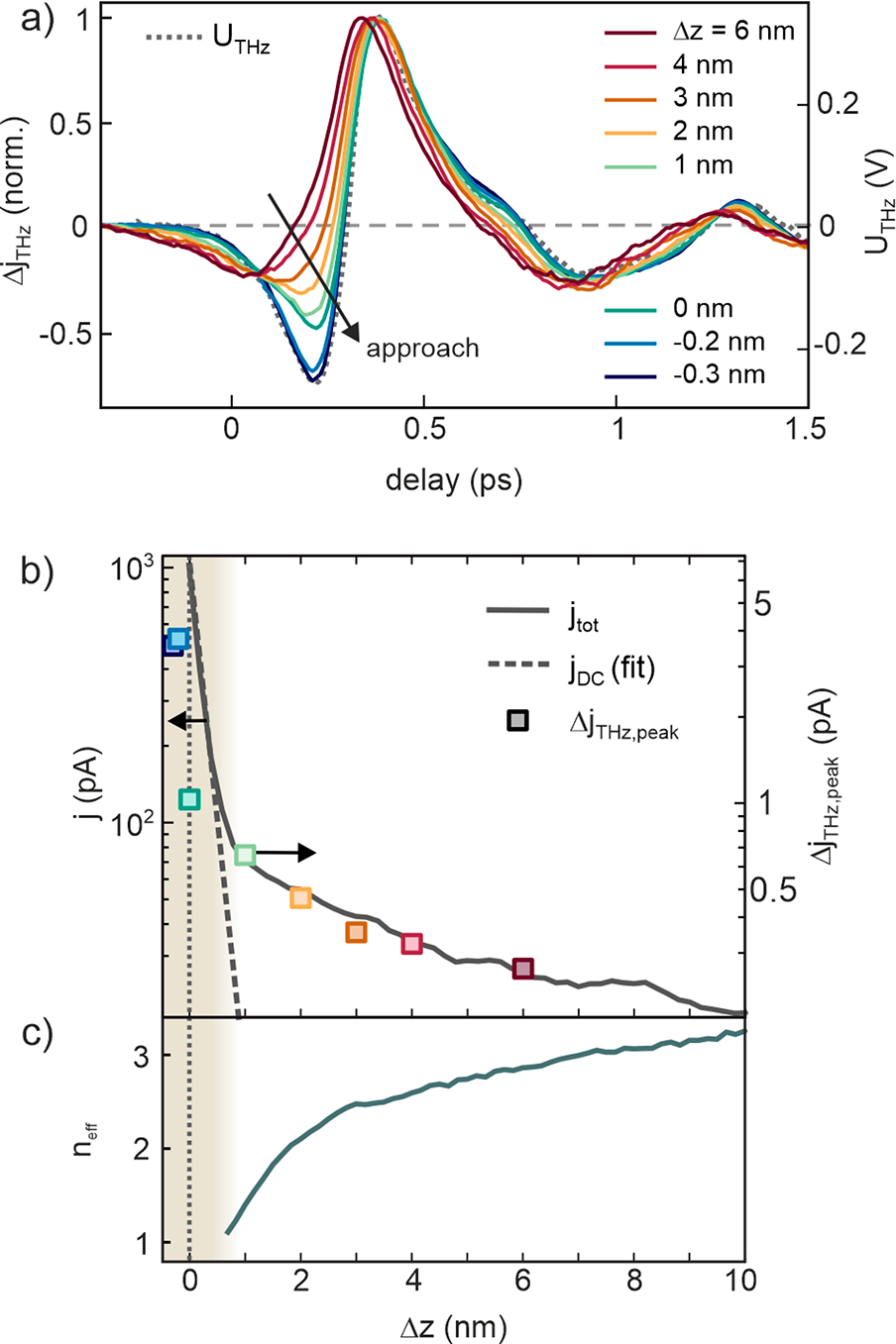
Gap size dependence of measured THz waveforms. (a) THz near-field waveforms measured against relative gap distance  from the set point. The THz waveform gradually transforms into the original THz bias waveform at short gap distances. (b) Gap size dependence of the total current (left *y*-axis) and THz-induced change of the photocurrent (right *y*-axis, peak value). Recovery of the original THz bias waveform correlates with the onset of static tunneling (dashed line). (c) Effective nonlinearity  of the photocurrent versus relative gap distance (set point 1 nA and 10 V, laser power 2.1 mW).

To examine the role of thermalized hot electrons for photoinduced tunneling in STM, we measure the THz waveform versus tip–sample distance as plotted in Figure 4a. The NIR power is set to a value that yields significant waveform deformations due to delayed thermal photocurrents at large tip–sample distances. The zero position  nm is defined by the STM set point of  V and  nA, at which the current is dominated by static tunneling. The relative gap distance is varied in the range between  and  nm (negative values mean smaller gap size). We can roughly estimate the absolute tip–sample distance by fitting the decay of the static current (dashed line in Figure 4b) and extrapolating to the quantum conductance , which yields  nm for  nm, which is reasonable at our conditions. As demonstrated previously, the THz bias does not depend on gap size;^11,30^ therefore, the THz bias measured at large gap distance (gray dashed line) applies to all distances. We note that “static” rectified THz currents are negligible due to the small THz bias amplitude and the comparably large gap distance when operating at 10 V bias. As discussed in the Supporting Information, our measurements are not affected by quasi-static or transient (pulse-to-pulse) thermal expansion of the STM tip due to pulsed laser illumination. Starting from a distorted waveform at  nm, the waveform continuously transforms such that it reproduces the original waveform of the THz bias at the smallest gap size. Complete vanishing of the thermal waveform distortions correlates with the onset of static tunneling, as evident from the distance dependence of the current plotted in Figure 4b. This is surprising, as naively one would expect a slower current decay due to increased tunneling at lower energies, for which the occupation and current decay times are larger than at the high-energy tail of the Fermi distribution. In addition, ultrafast heating will be enhanced at nanometer distances, because the optical field, and hence the absorbed power, are inversely proportional to the gap size. The dependence of the waveform deformations on the gap distance, which is distinctly different from the dependence on laser power shown in Figure 2b, thus clearly indicates that instantaneous current contributions must exceed thermionic tunneling at reduced gap size close to the static tunneling regime. Figure 4c shows the effective nonlinearity extracted from current-distance curves measured at varying laser pulse energy (details are described in section S6 in the Supporting Information). Whereas at large tip–sample distances the photocurrent exhibits a nonlinearity of , the nonlinearity continuously decreases with decreasing , indicating that photoassisted tunneling of lower orders dominates at small gap sizes.

**Figure 5:**
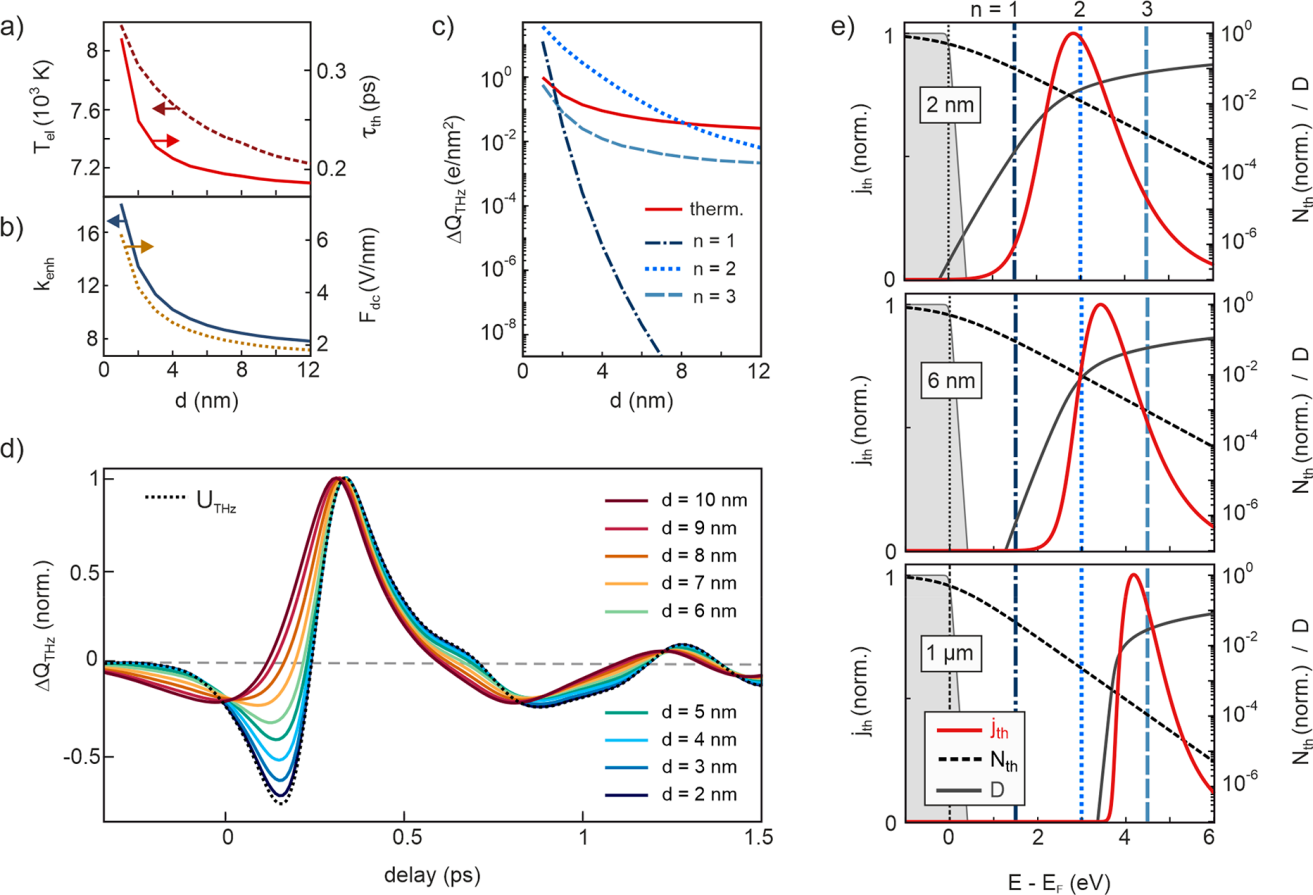
Simulation of photocurrent channels and THz waveforms versus tip–sample distance. (a) Distance scaling of electron peak temperature (left) and decay time of the thermionic current (right). (b) Distance scaling of optical field enhancement (left) and dc field (right). (c) Dependence of the THz-induced charge density  on gap size for thermionic tunneling (red) and nonthermal photoassisted tunneling at energies  (dashed) at the peak of the THz pulse. (d) THz waveforms calculated for the combined photocurrent channels shown in (c) at varying gap distance. (e) Energy distribution curves of the thermionic current (solid red, left *y*-axis) at delay  fs after photoexcitation for three gap distances 1 μm (bottom), 6 nm (middle), and 2 nm (top). The black dashed curves show the corresponding Fermi–Dirac distributions (*N*_th_, log-scale) with peak temperatures of  K at 1 μm,  K at 6 nm, and  K at 2 nm. The black solid curve shows the transmission probability  in the absence of the THz field. The blue vertical lines mark the positions of the nonthermal photocurrent channels . The gray shaded area shows the Fermi-Dirac distribution at room temperature.

The observations in Figure 4 suggest that at close distances the instantaneous tunneling of nonthermal electrons competes with delayed thermionic tunneling from the laser-excited STM tip. To corroborate this assumption, we simulate THz waveforms for varying gap distances . With decreasing , the dc field, THz field, and optical field at the tip apex increase, as do the absorbed power and hence the electronic temperature inside the tip. Figure 5a,b shows the distance scaling of , , and the optical field enhancement at the tip apex, together with the decay time  of the ultrafast thermionic current. The longer decay times  at reduced gap distances result from the higher electron temperatures and an increasing contribution from electrons tunneling at lower energies. Figure 5c compares the gap size dependence of  due to thermal and nonthermal electrons of orders , respectively. The THz waveforms calculated from the superposition of those currents are plotted as a function of  in Figure 5d, which reproduces well the experimental observations in Figure 4a. We can thus explain the gap size dependence of the measured THz waveforms by the competition between delayed thermal and prompt nonthermal tunneling from the photoexcited STM tip. The distinctive THz waveform changes can be understood from the distance-dependent mixture of thermal and nonthermal photocurrents, whose relative contributions vary with gap size according to their specific tunneling probabilities and nonlinear dependence on the local laser field, as reflected in the different slopes in Figure 5c. Whereas the nonthermal contribution from  increases mainly due to its nonlinear dependence on local laser intensity, the lower order contributions  and, in particular,  increase rapidly at reduced gap size due to their steeply increasing tunneling rates. In contrast, the source term of electronic heating in the TTM scales linearly with laser intensity. Moreover, the thermionic current is integrated over energy and thus exhibits an overall reduced sensitivity to the barrier compared to low-order nonthermal tunneling channels. This becomes apparent from the energy distribution of  plotted in Figure 5e together with the corresponding thermal electron occupation  and transmission probability  for three gap sizes at  fs. Even though the peak of the thermionic current distribution shifts to lower energies and develops a significant low-energy tail at nanometer gap distances, a majority of thermalized electrons are emitted at energies ∼2 eV above  and, hence, experiences a reduced sensitivity to the THz field compared to the nonthermal electrons excited into channel . It is the nonlinear laser intensity dependence of channels  on the one hand and the higher sensitivity of low-order channels to the tunneling barrier on the other hand that cause the instantaneous nonthermal currents to exceed the delayed thermionic current at nanometer gap sizes. Our findings clearly show the pronounced nonthermal and prompt character of hot electron-induced tunneling from a photoexcited STM tip.

Finally, we note that our simple model can reproduce the data reasonably well without calculation of the full nonthermal steplike distribution. The experimental data, however, shows a waveform change, within a distance variation of only 1 Å close to the set point, more rapid than that observed in the simulations. This indicates that our model might underestimate nonthermal hot electron tunneling close to the Fermi level, which exhibits an even higher sensitivity to the tip–sample distance. Further improvement of the agreement between the experimental and simulated results could be obtained by extending our model by (i) the simulation of the electron thermalization process, (ii) the inclusion of the full nonthermal distribution, and (iii) replacing the Fowler–Nordheim barrier by the Simmons barrier,^52^ which is more appropriate at nanometer gap sizes. We expect that more detailed spectroscopic information about the nonthermal distributions might be obtained in future experiments by variation of the dc and THz bias. Moreover, it may be possible to measure the electron thermalization process and its effect on tunneling by using shorter THz transients to increase the time resolution.

## Conclusions

In conclusion, we investigated the competition between ultrafast thermal and nonthermal photocurrents inside a laser-excited STM junction via phase-resolved sampling of broadband THz transients. Our results reveal that hot electron tunneling from a laser-excited STM tip is dominated by nonthermal electron distributions, accompanied by delayed tunneling of thermalized hot electrons. At large gap sizes, at which photoinduced tunneling through the barrier is negligible, thermalized hot electrons dominate the THz-induced photocurrent from a tungsten tip excited at high laser intensities. However, at small gap distance, this current contribution is exceeded by nonthermal currents as soon as the probability for photoassisted tunneling at low energies below the vacuum level increases appreciably. We infer the markedly nonthermal character of the photoexcited electron distribution from the distinctly different durations of the thermal and nonthermal photocurrents. Prompt nonthermal tunneling is followed by a thermionic photocurrent that can be delayed by several 100 fs, as determined by the time at which the laser-excited STM tip reaches its peak electronic temperature, which can increase up to several thousand Kelvin. The weak THz field serves as a highly sensitive and selective probe for photoinduced tunneling currents in ultrafast laser-excited STM, which is a unique capability of THz-gated STM. Detailed information about the phase and amplitude of the THz bias allows one to extract the ultrafast dynamics of hot electrons on time scales much shorter than a single THz cycle even in nonresonant systems like metals or semimetals. Our results provide detailed microscopic insight into the nonequilibrium dynamics of hot electrons in a laser-excited STM tip, which is of general relevance to experiments that aim to study photophysical or photochemical processes on ultrafast time scales with pulsed-laser excited STM. We envision that extension of our approach by the imaging capabilities of STM will allow for the spatiotemporal investigation of ultrafast carrier dynamics at photoexcited surfaces on Angstrom length and femtosecond THz-subcycle time scales.

## Methods

Measurements are recorded with a customized STM from Unisoku (USM-1400 with Nanonis SPM controller) operated at room temperature and under ultrahigh vacuum conditions (base pressure of <5 × 10^–10^ mbar). The spring-loaded STM platform is equipped with two off-axis parabolic mirrors (PM, 1 bare Au and 1 protected Ag, 1 in. diameter, 35 mm focal length) for illumination with a broadband NIR laser and broadband THz pulses, respectively. Both beams are incident to the tip axis at an angle of 68° and are polarized along the tip axis. The THz beam enters the UHV chamber through a 500 μm thick diamond window and is focused by the Au PM. The Ag PM is used to focus the NIR pulses for photoexcitation of the STM junction. Precise focus alignment on the tip apex is ensured by precise positioning of the PMs in UHV, which are motorized and can be moved in the *x*-, *y*-, and *z*-directions (Attocube GmbH). The tip position is fixed, and the sample is moved for coarse motion and scanning. The dc bias is applied to the sample and the current is collected from the grounded tip. The current preamplifier (Femto DLPCA) is operated at a gain of 10^9^ V/A at 1 kHz bandwidth. A mechanical chopper operated at a frequency of 607 Hz modulates the power of the NIR laser beam used for THz generation, and lock-in detection is used to detect the THz-induced current. Repeated cycles of Ar^+^ sputtering and annealing up to 670 K were conducted to clean the Ag(111) sample before measuring. Electrochemically etched tungsten tips are transferred to UHV immediately after etching.

The laser system is a broadband optical parametric chirped-pulse amplifier (Venteon OPCPA, Laser Quantum) delivering NIR laser pulses of 10 fs duration at 800 nm center wavelength and with 3 μJ energy (2 μJ are available for the THz-STM setup) at  MHz repetition rate. Part of the laser power is used for the generation of single-cycle THz pulses from a spintronic THz emitter (STE, 5.8 nm thick W/CoFeB/Pt trilayer on 500 μm sapphire substrate)^53^ excited at normal incidence in transmission geometry. A motorized translation stage is used to control the delay between the THz pulses and the NIR laser pulses used for photoexcitation of the STM. Part of the NIR laser pulses can be overlapped collinearly with the THz beam for THz pulse characterization using electro-optic sampling, as well as for precise THz beam alignment inside the STM. Peak laser intensities are calculated assuming a sech^2^-shaped laser pulse with a Gaussian beam profile, for which the peak intensity is determined as , where  is the fwhm laser pulse duration and  is the peak fluence in the center of the Gaussian beam with waist , and  is the pulse energy at a given laser power . A beam waist of  μm was used in the calculation, which was measured inside the STM under ambient conditions after the same alignment procedure we follow under normal operation conditions.

Numerical simulations are carried out using COMSOL Multiphysics 5.6., which provides the required values of the dc field, tip-enhanced optical field, and electronic temperature evolution inside the tip. The photoinduced thermal and nonthermal currents and their THz-induced change (THz waveforms) are simulated using custom Python scripts. Details about the model are described in the Supporting Information.

## Supporting Information Available

Calibration of the THz bias, bias dependence of photocurrent-power scaling at large gap size, THz waveforms for a tip operating solely in the weak-field regime, theoretical model, SEM image of STM tip, power-dependent current-distance curves, discussion of thermal expansion (PDF)

## Abbreviations

STM: scanning tunneling microscope
THz: terahertz
TTM: two-temperature model
NIR: near-infrared
SEM: scanning electron microscopy
STE: spintronic THz emitter
