# Supplementary material for: Femtosecond Thermal and Nonthermal Hot Electron Tunneling Inside a Photoexcited Tunnel Junction
Source: ACS Nano. 2022 Aug 26;16(9):14479–89. doi: 10.1021/acsnano.2c04846 (PMC9527804; doi:10.1021/acsnano.2c04846)
Supplement: Supplementary file 1 — nn2c04846_si_001.pdf [file nn2c04846_si_001.pdf]

Supporting Information

Femtosecond Thermal and Nonthermal Hot Electron Tunneling Inside a Photoexcited Tunnel Junction

Natalia Martín Sabanés [1,2,†], Faruk Krecinic [1,†], Takashi Kumagai [1,3], Fabian Schulz [1], Martin Wolf [1] and Melanie Müller [1,]*

[1] Department of Physical Chemistry, Fritz Haber Institute of the Max Planck Society, Faradayweg 4-6, 14195 Berlin, Germany.

[2] IMDEA Nanoscience, Faraday 9, 28049 Madrid, Spain.

[3] Center for Mesoscopic Sciences, Institute for Molecular Science, 444-8585 Okazaki, Japan

[†] Equal contributions

[*] Corresponding author: m.mueller@fhi-berlin.mpg.de

Content

1. **Calibration of the THz bias**
2. **Bias dependence of photocurrent-power scaling at large gap size**
3. **THz waveforms for a tip operating solely in the weak-field regime**
4. **Theoretical model**
5. **SEM image of STM tip**
6. **Power-dependent current-distance curves**
7. **Discussion of thermal tip expansion**

1. Calibration of the THz bias

The THz voltage between the tip-sample junction can be calibrated if (i) the NIR-induced photocurrent emitted from the tip is instantaneous on the time scale of the THz field, and if (ii) the current-voltage dependence of the photocurrent is known. Figure S.1a) shows the photocurrent versus DC bias measured for the same tip condition as the data shown in Figure 2) in the main manuscript. The tip-sample distance is 1 µm and the incident laser intensity $I_{\mathrm{peak}} = 0.6x10^{11}$ W/cm^2 is low enough to ensure operation without thermionic current contributions. Due to kinetic excess energy of the emitted photoelectrons, we observe photocurrent from the tip also at moderate negative bias. At larger negative bias the photocurrent reverses sign and is dominated by photoemission from the Ag sample. Vice versa, photoelectrons from the sample might contribute to the current at moderate positive sample bias. Consequently, we record the THz waveform at 8 V DC bias. Due to the quasi-static nature of the THz field, the THz amplitude can be calibrated by dividing the THz-induced change of the photocurrent by the linear I-V slope obtained from the DC bias dependence. Figure S.1b) shows the retrieved THz bias waveform (see also Figure 2a)). The THz bias is calibrated routinely before each measurement.

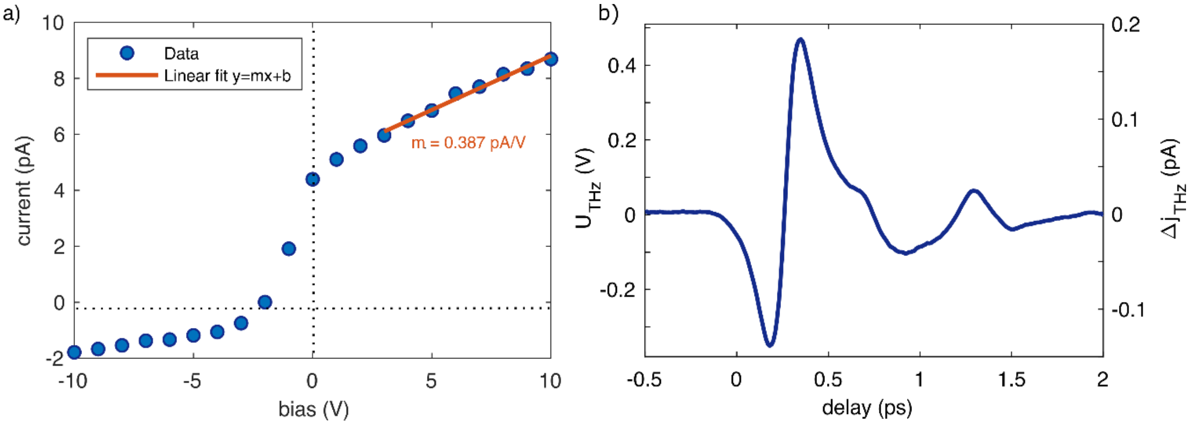

Figure S.1 *(a) Photocurrent-voltage curve used to calibrate the THz bias in Figure 2 in the main manuscript. (b) Calibrated THz bias (left y-axis) and corresponding THz-induced photocurrent change (right y-axis) measured at 8 V DC bias. (*$d = 1$ *µm and* $I_{peak} = 0.6x10^{11}$ *W/cm²)*

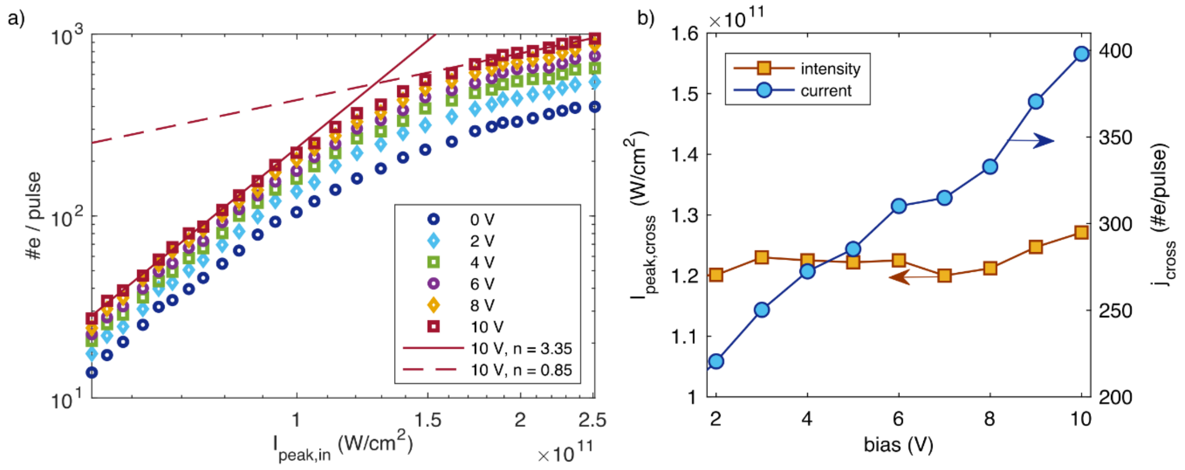

Figure S.2 *(a) Photocurrent versus NIR peak intensity at different DC bias measured at 1 μm tip-sample distance. Solid and dashed red lines are power law fits to the low- and high-power regimes shown exemplary for 10 V DC bias. (b) Laser intensity (left y-axis) and photocurrent (right y-axis) at which the transition from multiphoton scaling to nearly linear scaling occurs, defined as the crossing point between the power law fits of the two regimes (solid and dashed lines in (a)).*

2. Bias dependence of photocurrent-power scaling at large gap size

Figure S.2a) shows the dependence of the photocurrent on the incident laser intensity for DC bias in the range 0 V to 10 V measured at $d = 1$ μm for the tip condition used in Figure 2. We find that the transition from multiphoton scaling (power exponent $n \sim 3.4$) to a regime of nearly linear photocurrent scaling ($n \sim 0.9$) occurs at the same laser intensity for all DC bias, despite a 2x increase of the photocurrent from 0 V to 10 V, as plotted in Figure S.2b). This proves that space charge is not responsible for the observed saturation, but that we observe the transition to the strong-field regime[1]. This is reasonable considering the employed laser intensity and a Keldysh parameter of $\gamma \sim 2$ at the transition.

3. THz waveforms for a tip operating solely in the weak-field regime

It was argued recently that delayed photocurrents can originate from laser-driven inelastic re-scattering and re-emission of photoelectrons from a laser-excited tungsten tip[2]. In order to generate sufficiently delayed photocurrents, a significant amount of photoelectrons need to be driven back to the tip, re-enter the tip, undergo scattering processes inside the tip, and get re-emitted after a certain time delay. Especially in the presence of a static bias, the oscillating laser field needs to be strong enough to reverse an electron's trajectory and steer it back into

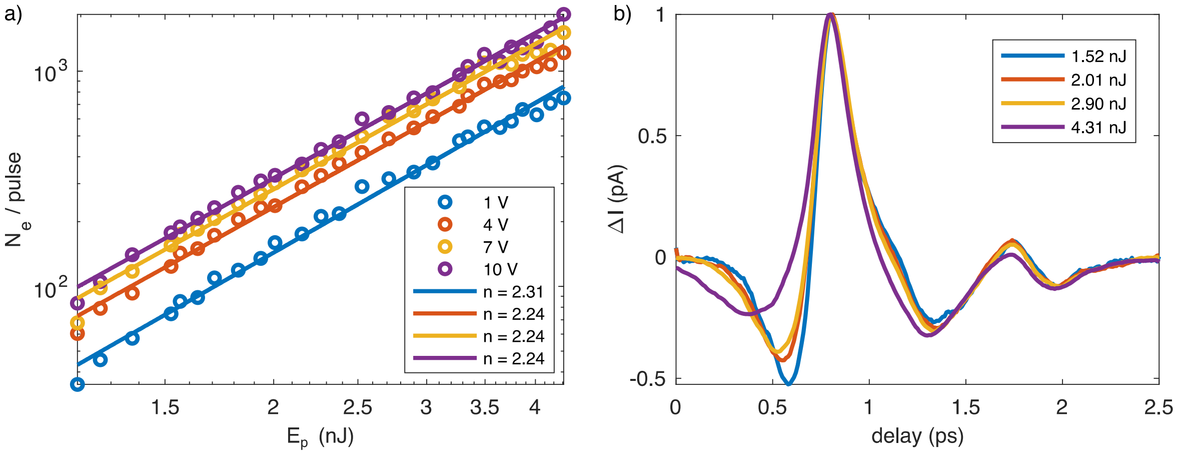

Figure S.3 *(a) Dependence of the photocurrent on the NIR pulse energy for a blunter tip, exhibiting multiphoton scaling for the entire pulse energy range. (b) Corresponding THz waveforms measured at four different powers (8 V DC bias, 1 μm tip-sample distance).*

the tip within less than an optical cycle. Hence, if operative, such source for delayed photocurrents can be expected to occur only in the strong-field regime of photoemission. We can exclude that electron scattering and subsequent re-emission is responsible for the observed THz waveform deformations, as for some tips we observe delayed photocurrents solely in the multiphoton photoemission regime. Figure S.3a) and S.3b) show the dependence of the photocurrent and the THz waveforms on the laser pulse energy for a blunter tip condition, respectively. In this case, strong THz waveform deformations of similar character as those in Figure 2 are observed purely in the multiphoton regime, as a nonlinear power scaling with constant slope $n = 2.24$ is observed in the investigated laser power range. This data is recorded from the same tip as used in Figures 2 and S.2, but after the tip condition has changed. We observe that (i) the photocurrent and its power scaling, (ii) the observation of a delayed photocurrent and the required threshold laser intensity, and (iii) the calibrated THz bias amplitude all correlate with the detailed nanoscale tip condition. This indicates that the nanoscale shape of the tip does not only affect the optical field enhancement but also the electron dynamics inside the apex considerably.

4. Theoretical model

THz waveforms due to thermal electron distributions are calculated by the following procedure: First, we calculate the enhanced near-infrared (NIR) laser field and absorbed

power density in the tip-sample junction using the RF-Module of COMSOL Multiphysics 5.6 by solving the time-harmonic wave equation in three dimensions. The tip-sample junction is excited by an incident NIR Gaussian laser beam with 2 μm waist and peak laser field F_{in}, whose magnitude is varied in a range close to the incident NIR laser field strength used in the experiments.

Second, we convolve the obtained optical power density inside the tip, S_a, with the ballistic transport range λ_b of electrons in tungsten.[3] Specifically, we convolve the absorbed power profile S_a with an exponentially decaying impulse response function[4] $\propto e^{-\lambda_b r}/r$, which yields the power density distribution after spatial redistribution of energy due to ballistic transport, S_b. This is performed within the COMSOL Multiphysics simulation environment inside the 3D geometry of the tip by solving the Helmholtz equation

$$\nabla^2 S_b - \lambda_b^2 S_b = S_a. \qquad (S1)$$

Figures S.4.1a) and S.4.1b) compare the spatial profiles of S_a and S_b for tip-sample distances of $d = 10$ nm and $d = 1$ nm, respectively. The effect of ballistic transport on the spatial profile of the absorbed power density is clearly evident from the comparison of S_a and S_b, which shows how ballistic transport spreads the energy deposited quasi-instantaneously inside a nanolocalized volumes such as an STM tip excited by an ultrafast laser pulse.

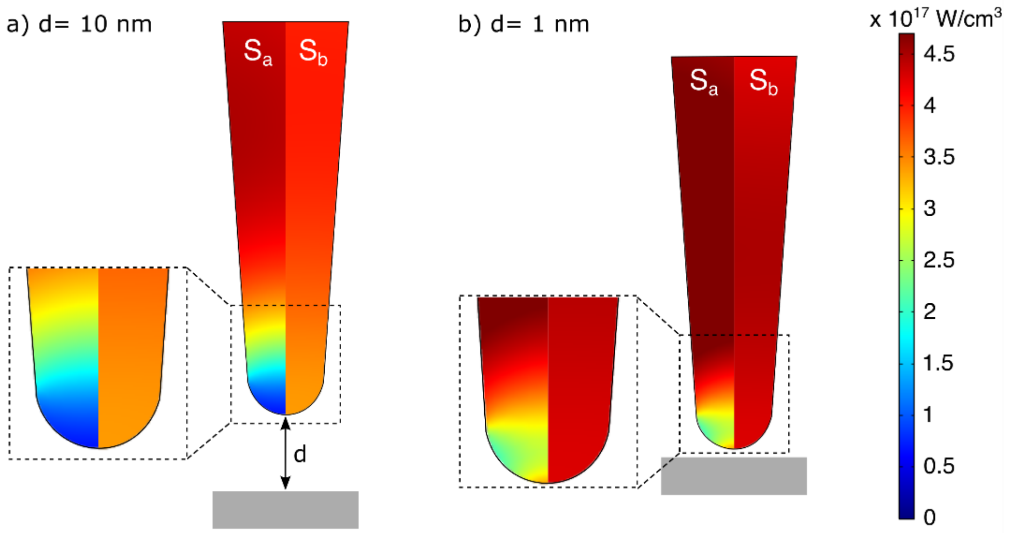

Figure S.4.1 *Spatial profiles of the absorbed power density inside the STM tip before (S_a) and after (S_b) spatial re-distribution of energy due to ballistic electron transport for tip-sample distances of (a) 10 nm and (b) 1 nm.*

Third, we obtain the temperature evolution of the electronic and phononic sub-systems from the two-temperature model (TTM) by solving the differential equations

$$C_{\mathrm{el}}\frac{\partial T_{\mathrm{el}}}{\partial t} = \boldsymbol{\nabla}(k_{\mathrm{el}}\boldsymbol{\nabla}T_{\mathrm{el}}) - G\big(T_{\mathrm{el}} - T_{\mathrm{ph}}\big) + S_{\mathrm{TTM}}(t) \tag{S2a}$$

$$C_{\mathrm{ph}}\frac{\partial T_{\mathrm{ph}}}{\partial t} = G\big(T_{\mathrm{el}} - T_{\mathrm{ph}}\big) \tag{$S2b$}$$

in three dimensions, where T is temperature, C is the specific heat capacity, k is the thermal conductivity, G is the electron-phonon coupling and the subscripts 'el' and 'ph' denote the electron and phonon sub-systems, respectively. C_{el} and k_{el} are considered temperature dependent and described by $C_{\mathrm{el}}(T_{\mathrm{el}}) = \gamma T_{\mathrm{el}}$ and $k(T_{\mathrm{el}}) = k_{\mathrm{eq}}(T_{\mathrm{el}}/T_{\mathrm{ph}})$, where γ is the electron heat capacity constant and k_{eq} is the electron thermal conductivity at equilibrium. Since it can be assumed that ballistic redistribution of the energy takes place quasi-instantaneously on the time scale of the THz field, we use the power density profile S_b to calculate the source term for the TTM,

$$S_{\mathrm{TTM}}(t) = S_b \exp\left[-2\frac{t - t_0}{t_p}\right]^2, \tag{$S3$}$$

where t_p is the NIR laser pulse duration. The system of partial differential equations is solved numerically using the following parameter values:[4] $\gamma = 137.3\ \mathrm{JK^{-2}m^{-3}}$, $k_{\mathrm{eq}} = 150\ \mathrm{Wm^{-1}K^{-1}}$, $G = 7.5 \times 10^{17}\ \mathrm{WK^{-1}m^{-3}}$, and $\mathrm{C_{ph}} = 2.58 \times 10^{6}\ \mathrm{JK^{-1}m^{-3}}$, which yields the electron and phonon temperatures at any point inside the tip. We note that the increase of the electronic temperature inside the tip is determined by the absorbed power density inside the tip, which does not only depend on the incident laser intensity but to a large extent also on the nanoscale tip geometry. Ideally, the experimentally unknown tip geometry would be a free parameter in our model. However, this is computationally very demanding even for the simple geometry used here. We therefore keep the tip geometry fixed and instead vary the incident laser intensity to adjust the absorbed power density and electronic temperature. Test simulations at varying apex size and opening angle show that thermionic currents and THz waveform distortions are observed for all tip geometries but at varying incident laser intensities, where generally higher intensities are required to reproduce the experimental results for larger tip apex radius and opening angles.

Last, the calculated electronic temperature evolution at the tip apex is used to simulate the emission of electrons using Eqs. (2) – (5) from the main manuscript. The DC field at the apex, F_{dc}, is obtained from electrostatic simulations of the DC electric field inside the biased tip-sample junction using the AC/DC module of COMSOL Multiphysics 5.6, where the same tip geometry is used for DC and RF simulations. The peak THz field is determined from the experimentally known ratio of DC bias and THz bias and the simulated DC field as $F_{\mathrm{THz}} = [U_{\mathrm{THz}}/U_{\mathrm{dc}}]F_{\mathrm{dc}}$. Finally, we calculate the THz-induced change of the emitted charge density ~~ultrafast thermionic current~~, $\Delta Q\not{j}_{\mathrm{th}}(\tau)$, as a function of NIR-THz time delay using Eq. (2).

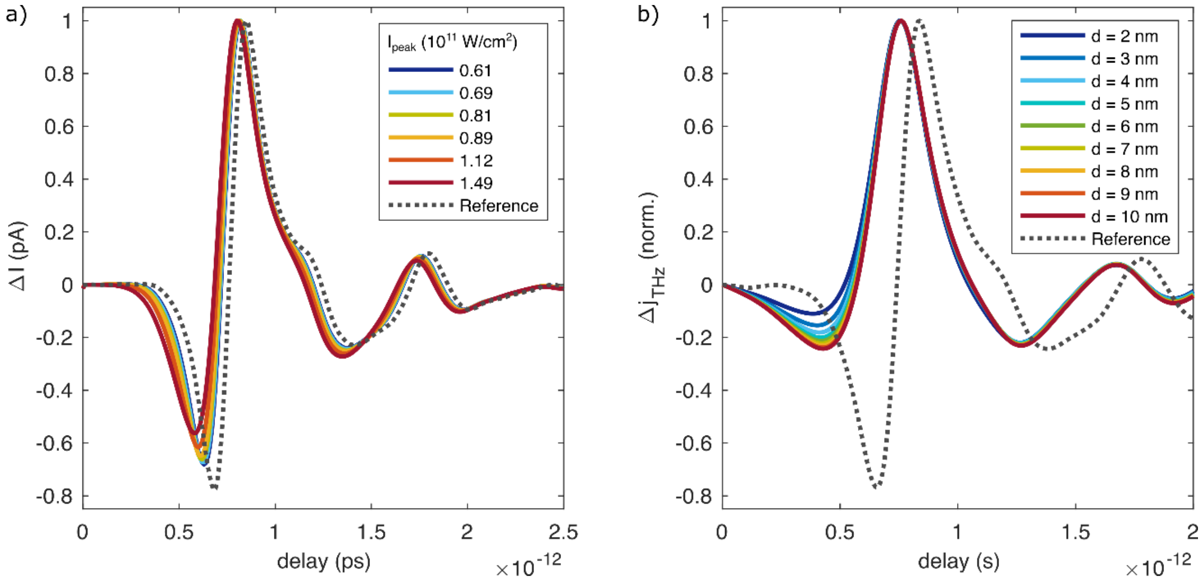

Figure S.4.2 *THz waveforms calculated for pure thermionic emission and their dependence on (a) incident laser intensity ($d = 1$ μm) and (b) tip-sample distance ($E_{in} = 1.4$ V/nm and $I_{peak} = 2.6 \times 10^{11}$ W/cm², respectively) at 8 V DC bias.*

Figure S.4.2a) shows the dependence of the THz waveform, which is simulated for pure thermionic currents, on the NIR laser intensity without contributions from nonthermal current due photoemission or photoassisted tunneling. The simulations reveal that 'pure thermionic' THz waveforms do not reproduce the original THz waveform exactly. Even at comparably low electron temperatures, at which the thermionic current density is very small, the calculated THz waveform exhibits typical low-pass filtered character, where the deformations of its shape originate predominantly from the slow decay, and the temporal

shift from the non-instantaneous increase of the electron temperature and the corresponding transient thermionic current.

Figure S.4.2b) shows THz waveforms simulated for pure thermionic currents at varying gap size. As expected from the distance dependence of the enhanced laser intensity and thus of the ultrafast heating of the electrons, which both increases inversely with gap size, the THz waveforms become more distorted at smaller gap size. This trend is further enhanced by the reduced barrier width at small gap sizes.

To calculate THz waveforms due to nonthermal electron distributions, we use the tip-enhanced laser field at the tip surface as the input for equation 6. The excitation parameter C_{ex} is initially adjusted such that the occupation in channel n decreases with increasing order by similar values as those reported in previous literature[5]. We note that because N_n has units of a number density (m^{-2}), the unit of C_{ex} will depend on the nonlinearity, just as the unit of a (non)linear absorption cross section depends on the nonlinearity of the process. The value of the scaling factor C_0 is adjusted such that the amplitude of the nonthermal current becomes large enough to compete with the thermal current. If C_0 is too small, the simulated waveforms are determined by pure thermionic currents, which does not yield the waveform scalings we measure. If C_0 is too large, the simulated waveforms originate solely from nonthermal currents, and no waveform distortions are observed at any laser intensity or gap distance. Because thermal and nonthermal currents are added, the unit of Q_{nonth} has to be the same as the charge density in eq. 2, i.e. Cm^{-2}. Since N_n has units of m^{-2} and D_n is dimensionless, the unit of C_0 will be C·s. The exact value of C_0 does not provide quantitative insight because we include only delta-like channels at $E_n = n\hbar\omega$ with $n = [1,2,3]$ to calculate the nonthermal current instead of taking into account the full nonthermal distribution, and hence underestimate the nonthermal current amplitude. Moreover, quantitative understanding of the nonthermal current amplitude, and hence C_{ex} and C_0, would require knowledge of all possible (linear and nonlinear) excitation channels from initial states $|i\rangle$ into final states $|f\rangle$ and calculation of the respective (nonlinear) transition rates, which is beyond the scope of this work.

5. SEM image of STM tip

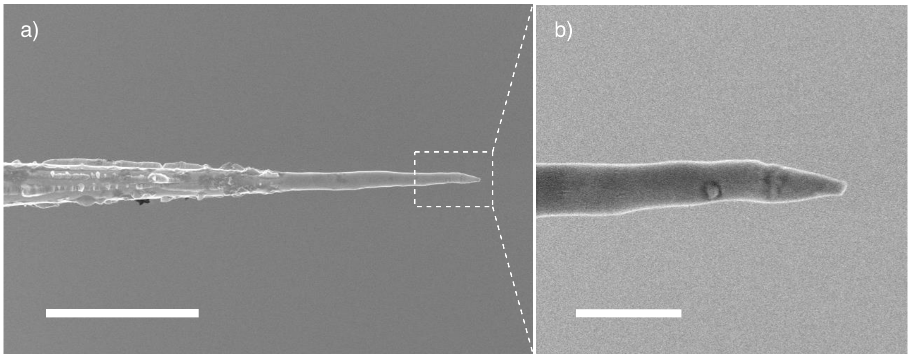

Figure S.5 *SEM images of the tungsten tip used in this work. Images are recorded after several weeks of tip usage inside the STM. Scale bars correspond to (a) 3 μm and (b) 500 nm. The area of the tip shaft with reduced surface roughness in (a) matches the approximate laser spot size.*

6. Power-dependent current-distance curves

In order to extract the nonlinearity of the photocurrent at nanometer gap distances, we measure photocurrent-distance curves at different laser powers. Figure S.6.1a) shows few examples of $j_{ph} - z$ curves over the power range used here. For all powers the curves overlap at the closest distances $\Delta z \lesssim 1.5$ nm and we observe a clear transition to the DC tunneling regime, even at the highest powers. At the set point, the current of 1 nA is an order of magnitude larger than the photocurrent observed outside but close to the DC tunneling range. We can thus assume that the set point distance is determined predominantly by the DC tunneling current. Fitting the DC current and subtracting this from the total current thus yields the distance scaling of the pure photocurrent. Figure S.6.1b) shows the laser power dependence of the resulting photocurrent and linear fits for distances in the range 1 nm to 12 nm. The retrieved nonlinearities are plotted versus relative gap distance in Figure 4c) in the main manuscript.

Even though at the set point the photocurrent contribution to the total current is small, the absolute tip position and gap distance will depend on the NIR laser power, as the feedback will compensate for the small but increasing photocurrent contribution at higher laser intensities. To estimate this effect, we extrapolate the fitted DC current at each power to the quantum conductance G_0, which reveals a difference of the absolute gap distance of ~0.5 nm between the highest and lowest power. Figure S.6.2a) shows the power dependence of the

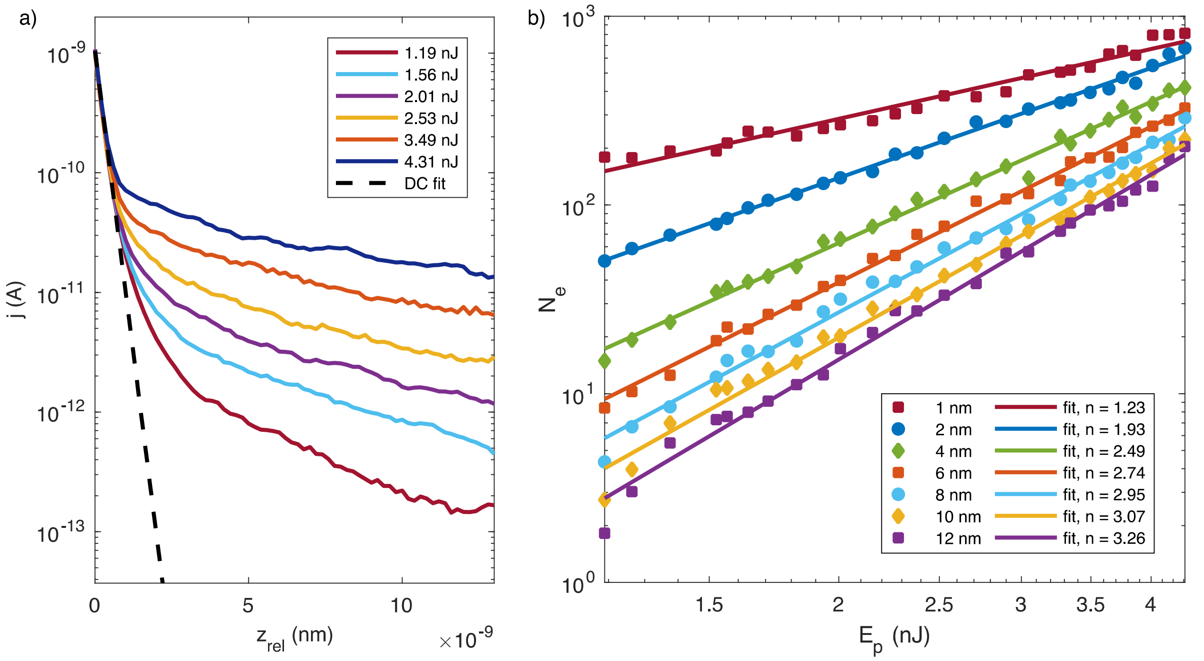

***Figure S.6.1** (a) Photocurrent-distance curves measured at different NIR laser powers. Zero relative distance is defined by the STM set point of 1 nA and 10 V. The black dashed line is a fit to the DC tunneling current, which dominates at small distances. (b) Laser power scaling of the photocurrent at different gap distances, as retrieved from vertical cuts of the $I_{ph} - z$ curves in (a) after subtraction of the DC current from the total current. Solid lines are power law fits to extract the effective nonlinearity n_{eff}.*

estimated absolute tip-sample distance d, revealing the set point correction due to the photocurrent contribution to the total current. Note that this is only a small fraction of the measured change in z versus power, plotted in Figure S.6.2b), which originates predominantly from thermal expansion. We checked that the 0.5 nm offset due to the photocurrent has negligible effect on the extracted nonlinearity in the investigated range at our conditions. We estimate this by shifting the $j_{ph} - z$ curves along the Δz-axis according to their difference in the absolute distance d. Figure S.6.2c) compares the effective nonlinearity retrieved from the DC-fit corrected, but unshifted $j_{ph} - z$ curves (blue, same as Figure 4c)) and the DC-fit corrected, but shifted and d-corrected curves (orange). We find that the ~0.5 nm change of the absolute tip-sample distance has insignificant effect on n_{eff} in the range $\Delta z > 1$ nm at our conditions. We are hesitant to conclude on the range $\Delta z < 1$ nm as this is difficult to fit reliably due to the large scatter of the data in the current-power curves originating from subtraction of the fitted DC current. It is clear, though, that the

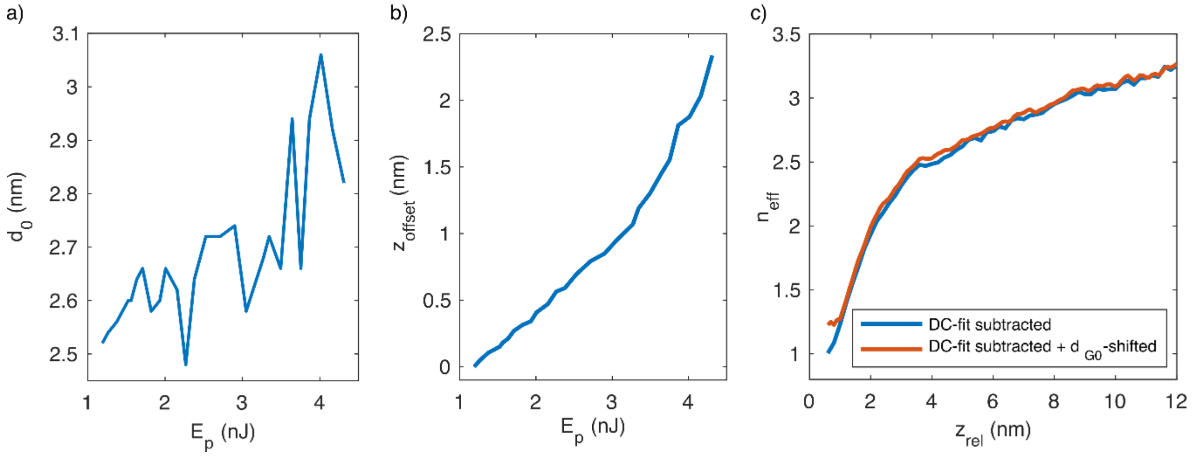

Figure S.6.2 *(a) Estimated absolute gap distance at the set point as a function of laser pulse energy, obtained by extrapolating the fitted DC current to the quantum conductance G_0. The contribution of the photocurrent to the total current results in* ~ 0.5 *nm variation of the gap distance at the set point between the lowest and highest powers used. (b) Offset of the z-piezo versus incident pulse energy, which originates predominantly from steady-state thermal expansion of the tip, which is significantly larger than the z-correction due to an increasing contribution of photocurrent to the total current. (c) Change of effective nonlinearity with gap size. Blue curve: only the DC current is subtracted from the* $I_{ph} - z$ *curves (Figure S.6.1a); Red curve:* $I_{ph} - z$ *curves are additionally shifted by the gap distance change shown in (a) to account for the z-correction at the set point due to the increase of the photocurrent with increasing laser power.*

effective nonlinearity decreases significantly and approaches the range $n_{\text{eff}}{\sim}1$ at the closest distances. This corroborates the assumption that a significant amount of electrons in channel $n = 1$ at low energies contributes to the photocurrent and is responsible for the very fast transition to the original THz waveform within a few Angstrom distance change.

7. Discussion of thermal tip expansion

When the STM tip is illuminated with a pulsed laser, two types of thermal expansion can occur which can be distinguished from their different time scales: (i) quasi-static expansion and (ii) transient (pulse-to-pulse) expansion. Quasi-static 'cw-like' expansion originates from the steady-state temperature rise of the tip due to absorption of the laser light. It occurs on time scales slower than heat transport inside the STM tip[6] (> µs), at which the tip movement follows 'slow' variations due to for example laser power fluctuations or interferometric power modulations. In contrast, transient tip expansion occurs due to the pulse train structure of ultrashort lasers and the concurrent modulation of the absorbed laser energy and hence tip temperature at the laser repetition rate.

At large gap sizes (Figures 2 and S1-S3), both types of thermal expansion have negligible effect on the absolute gap distance, which is much larger than the expected thermal expansion of few nm. However, at small gap distances close to the STM set point (Figure 4), the quasi-static thermal expansion becomes comparable to the gap size (as observed in Figure S.6.2) and will affect the current. To ensure that quasi-static expansion does not affect our measurements, we wait for the z-piezo position to stabilize after switching on or changing the laser power before recording a measurement. In addition, to ensure that residual thermal drifts do not affect the waveform measurements at close distances, the data is recorded with an automated measurement script, which turns on the STM feedback at each delay point before the tip is moved from the set point to the desired distance at which the THz-induced signal is recorded. This ensures a constant absolute gap distance at each delay, provided that the illumination is stable. Hence, steady-state thermal expansion does not affect our measurements. We note that very good long-term stability of the laser illumination, i.e., high laser power stability and beam pointing stability, is absolutely necessary for these measurements.

Transient tip expansion can be a problem (i) if lock-in detection at the laser repetition rate is used, (ii) if the dynamics under investigation occurs on time scales similar to the time it takes the tip to expand after absorption of a single laser pulse, and/or (iii) if a mechanical contact between tip and sample is formed due to the periodic power modulation. Transient thermal expansion occurs on 100's ps to ns time scales and is thus slow compared to the time window we investigate, during which no significant heat transport has occurred yet. Moreover, the magnitude of expansion decreases with increasing laser repetition rate and with decreasing laser spot size and power.[7,8] Comparison with previously reported experimental and theoretical values shows that we do not expect contact formation at our experimental conditions of comparably large distances and high repetition rates. This is experimentally corroborated by the very good long-term-stability (several hours) of the photocurrent and THz waveforms we measure, and by the fact that we do not find surface modifications at the sample that could originate from contact formation. Hence, our experiment should not be affected by modulation of the gap size due transient thermal expansion.

References

(1) Bormann, R.; Gulde, M.; Weismann, A.; Yalunin, S.; Ropers, C. Tip-Enhanced Strong-Field Photoemission. *Phys. Rev. Lett.* **2010**, *105*, 147601. https://doi.org/10.1103/PhysRevLett.105.147601.

(2) Yanagisawa, H.; Schnepp, S.; Hafner, C.; Hengsberger, M.; Kim, D. E.; Kling, M. F.; Landsman, A.; Gallmann, L.; Osterwalder, J. Delayed Electron Emission in Strong-Field Driven Tunnelling from a Metallic Nanotip in the Multi-Electron Regime. *Sci. Rep.* **2016**, *6*, 35877. https://doi.org/10.1038/srep35877.

(3) Choi, D.; Kim, C. S.; Naveh, D.; Chung, S.; Warren, A. P.; Nuhfer, N. T.; Toney, M. F.; Coffey, K. R.; Barmak, K. Electron Mean Free Path of Tungsten and the Electrical Resistivity of Epitaxial (110) Tungsten Films. *Phys. Rev. B - Condens. Matter Mater. Phys.* **2012**, *86*, 045432. https://doi.org/10.1103/PHYSREVB.86.045432.

(4) Lin, Z.; Zhigilei, L. V.; Celli, V. Electron-Phonon Coupling and Electron Heat Capacity of Metals under Conditions of Strong Electron-Phonon Nonequilibrium. *Phys. Rev. B - Condens. Matter Mater. Phys.* **2008**, *77*, 075133. https://doi.org/10.1103/PHYSREVB.77.075133.

(5) Yanagisawa, H.; Hengsberger, M.; Leuenberger, D.; Klöckner, M.; Hafner, C.; Greber, T.; Osterwalder, J. Energy Distribution Curves of Ultrafast Laser-Induced Field Emission and Their Implications for Electron Dynamics. *Phys. Rev. Lett.* **2011**, *107*, 1–5. https://doi.org/10.1103/PhysRevLett.107.087601.

(6) Gerstner, V.; Thon, a.; Pfeiffer, W. Thermal Effects in Pulsed Laser Assisted Scanning Tunneling Microscopy. *J. Appl. Phys.* **2000**, *87*, 2574. https://doi.org/10.1063/1.372221.

(7) Grafström, S.; Schuller, P.; Kowalski, J.; Neumann, R. Thermal Expansion of Scanning Tunneling Microscopy Tips under Laser Illumination. *J. Appl. Phys.* **1998**, *83*, 3453.

(8) Xie, N.; Gong, H.; Yan, S.; Zhao, J.; Shan, X.; Guo, Y.; Sun, Q.; Lu, X. Tip Expansion in a Laser Assisted Scanning Tunneling Microscope. *Appl. Phys. Lett.* **2012**, *101*, 213104. https://doi.org/10.1063/1.4767877.
